# Influence of non-directional errors in anthropometric measurements and age estimation on anthropometric prevalence indicators

**DOI:** 10.1371/journal.pone.0304131

**Published:** 2024-09-04

**Authors:** Joseph M. Grange, Nancy B. Mock, Shalean M. Collins

**Affiliations:** 1 United States Agency for International Development, Washington, D.C, United States of America; 2 Environmental Incentives, South Lake Tahoe, California, United States of America; 3 School of Public Health and Tropical Medicine, Tulane University, New Orleans, Louisiana, United States of America; Mahidol University, THAILAND

## Abstract

Anthropometric prevalence indicators such as stunting, wasting, and underweight are widely-used population-level tools used to track trends in childhood nutrition. Threats to the validity of these data can lead to erroneous decision making and improper allocation of finite resources intended to support some of the world’s most vulnerable populations. It has been demonstrated previously that aggregated prevalence rates for these indicators can be highly sensitive to biases in the presence of non-directional measurement errors, but the quantitative relationship between the contributing factors and the scale of this bias has not been fully described. In this work, a Monte Carlo simulation exercise was performed to generate high-statistics z-score distributions with a wide range of mean and standard deviation parameters relevant to the populations in low- and middle-income countries (LMIC). With the important assumption that the distribution’s standard deviation should be close to 1.0 in the absence of non-directional measurement errors, the shift in prevalence rate due to this common challenge is calculated and explored. Assuming access to a given z-score distribution’s mean and standard deviation values, this relationship can be used to evaluate the potential scale of prevalence bias for both historical and modern anthropometric indicator results. As a demonstration of the efficacy of this exercise, the bias scale for a set of 21 child anthropometry datasets collected in LMIC contexts is presented.

## Introduction

Childhood growth indicators are an important tool to assess the trajectory of nutritional and other environmental characteristics of the youngest members of vulnerable communities. Over the last decade or so, the global community has coalesced around the stunting indicator (height-for-age z-score ≤ -2 SD) as a critical target for measuring progress in these areas. More recently, an important debate has challenged the causal pathways, and therefore the appropriate use of, this indicator [1, 2]. However, independent of its use, a reasonable understanding of the accuracy of this indicator and other anthropometric metrics such as wasting and underweight in individual contexts will always be an important factor in its interpretation.

Several factors contribute to reduced child anthropometric and nutrition data quality. Height and length, specifically, are particularly fallible. Recumbent boards are recommended as standard infant length measurement tools until 24 months of age [3], but are often unavailable [4, 5] or damaged [6], and are prone to measurement error [7]. Error can also be introduced if children are incorrectly assessed using standing height in place of recumbent length [3]. A correction factor of ±0.7cm can be applied to correct for this difference [3], but if not used can result in a misinterpretation of child size [8]. Recumbent length measurements can be difficult to complete due to child movement [6], knee bending [6, 9], and caregiver apprehension, which can result in infants not being correctly undressed for measuring [6].

For children 24 months and older, non-recumbent height measurement tools, such as wall-mounted stadiometers or growth charts are used [3], although these are also prone to error. Wall-mounted stadiometers have shown to produce an error range of (98.7–101.1cm) in one study, which was similar to the error range reported in the same study for wall-mounted growth charts (90.0–105.2cm) [10]. This error is substantial, considering that child growth can range from -1.5–2 cm for children 12–24 months experiencing drought conditions [11] to +5 cm per year among older children experiencing normal conditions [5, 12]. Standing height is also prone to error if children are wearing shoes [3, 5], have hairstyles or accessories that increase height [3], if heels and posterior body are not touching a wall [3], or if the child is not level with the Frankfurt plane [13].

Across all anthropometric measures, enumerator experience, training, and ability are critical in ensuring correct measurement and ultimately reducing error. In an inpatient pediatric unit study, enumerators cited a number of concerns with using wooden recumbent boards, such as fear of harming or traumatizing children, bulkiness and difficulty using equipment, perceptions of uncleanliness, and worries about inaccuracy of reporting [14]. These concerns, in addition to standard measurement error, resulted in approximately three percent of height- for-age z-scores reported by enumerators being implausible [14]. Additionally, enumerator measurement repeatability has shown to be difficult across individuals [10, 15], such that inter-observer technical error can present up to -0.37cm differences in recumbent length between expert anthropometrists and trained enumerators [7] and between 0.04 and 2.58cm between trained enumerators for recumbent length and standing height [16]. A randomized control trial across multiple primary care centers in the United States found only 30% of height measurements to be accurate between enumerators [5].

Children under five years old are classified as stunted if their height-for-age falls two or more standard deviations below the expected value for a healthy individual of their age and sex. There may be no biological basis for the widely- agreed-upon -2SD threshold, and despite its use for clinical diagnoses, the cutoff is largely arbitrary [17] and the risk of undesirable outcomes does not occur after the cutoff point [17, 18]. Indeed, studies have indicated that even mild to moderate malnutrition among children can result in increased risk of mortality [19, 20] and adverse developmental impacts [21] without clear evidence of an inflection point [20].

Beyond errors due to length and height measurement, age measurement can also introduce errors into reported length-for-age and height-for-age z-scores [22]. Due to reduced administrative capacity, local calendars and other factors, high quality age data in many low-resource settings can be challenging to achieve. One analysis estimates that globally only 65% of children under five have had their births registered [23], with nationally-representative surveys in some contexts reporting non-response rates up to 30% for child age [24]. In vulnerable communities, the number is likely much higher. Other aspects of the relationship between age and stunting data quality in LMIC contexts have been investigated in previous literature [25–31].

In contexts with lower rates of healthcare center deliveries, without accurate birth data, or with non-Gregorian perceptions of months and years, child age can be based on estimates centered around memorable events [24], overestimated based on the age children will be at their upcoming birthday [32], rounded to “0” or “5” ages, or at 12, 24, and 36 months, also known as “age heaping” [32–34], or birth year can be subtracted from the current year without accounting for specific dates [32]. Age misreporting can introduce significant systematic error [24]. Enumerator bias and reduced maternal literacy can also contribute to error in reporting child age, with one study indicating a difference of up to four months between interviewers [26].

Measurement errors generally fall into two categories: directional and non-directional. Every real-world source of measurement error is likely a mix of these broad groupings. For example, in colder climates children are more likely to wear bulky clothing that will bias weight values higher compared to the truth. As a consequence, the mean value in the weight-for-age and weight-for-height distribution will erroneously increase. If all children were to wear precisely the same amount of clothing, this measurement error would be purely directional. Of course there will be a diversity in clothing worn by individual children, and this results in a variety of weight biases. This adds a non-directional component of measurement error, and therefore the standard deviation in the weight-for-age and weight-for-height distributions will spuriously increase. In this example children wearing relatively heavy clothing introduces a *directional* measurement error which influences the distribution’s *mean value*, while varying bulk of clothing results in *non-directional* measurement error which will artificially inflate the distribution’s standard deviation.

Therefore, indicators that track only a distribution’s mean value are not affected by the presence of non-directional measurement errors. However, it will be shown that because anthropometric indicator prevalence rates are defined by the proportion of observations that exceed a certain threshold, non-directional measurement errors can significantly influence these rates.

Through simulation, this work quantifies how non-directional measurement error, regardless of variable source including height, age, or weight, can lead to systematic biases in anthropometric prevalence rates. While the influence of measurement error in anthropometric indicators has been documented elsewhere, to the authors’ knowledge this is the first quantitative investigation of the relationship between a z-score distribution’s mean, standard deviation, and range of plausible biases in calculated indicator prevalence. Also presented is a case study investigation of these bias estimates present in 21 surveys collected in LMIC environments and an exploration of the data quality that likely led to these biases.

## Methods

In this work, the effects of non-directional measurement error on childhood anthropometric prevalence indicators are evaluated through their consequence of inflating the z-score distribution’s standard deviation value. To this end, a Monte Carlo simulation exercise was performed to test the relationship between a normal distribution’s standard deviation and the rate of observations falling below a threshold value (in the case of childhood anthropometry, -2.0). It is readily apparent that the *mean* of the distribution also influences the calculated prevalence rate. Many normal distributions were simulated in an uniformly-spaced grid of mean and standard deviation parameters relevant to historical LMIC anthropometric data and symmetric around the special mean value of -2.0. The parameter ranges used are standard deviation values in {0.8,2.0} and mean values in {-4.5,0.5}. For each of these normal distributions representing child-level z-score survey data, the prevalence of observations falling below -2.0 was calculated. The normal distributions were simulated with 500,000 observations each.

Assuming a given z-score distribution is sufficiently normal, this relationship is adequate to estimate the prevalence rate for any childhood anthropometric indicator with mean and standard deviation values in the parameter range examined. But, like any measurement, the z-score distribution empirically determined by the research team a priori is some unknown combination of the genuine distribution and measurement error. However, some error artifacts are observable in hindsight and can help identify features that are likely the result of measurement error. For example, it is generally understood that standard deviation values for anthropometric distributions collected in high-quality conditions including intensive training and supervision should fall between 0.8 and 1.2 [25]. Therefore, any distribution with standard deviation significantly higher than 1.2 was likely influenced by non-directional measurement error. By taking the midpoint of this range (i.e., 1.0), the bias in prevalence due to non-directional measurement error can be estimated by taking the difference between the prevalence for a given mean and inflated standard deviation value and the prevalence at the same mean value but with a standard deviation at the more realistic value of 1.0. This is the primary result of this work and will allow practitioners to estimate the scale of bias present in childhood nutrition indicator data including stunting, wasting and underweight. Important assumptions to note include that the z-score distribution is normal, that the standard deviation value *would have* been close to 1.0 the the absence of non-directional measurement error, and that the presence of directional error is not large.

While these tests reveal the mathematical relationships between these parameters, it can be pedagogically useful to study a few of these normal distributions in greater detail. To better understand the mechanism behind the three-way relationship between a normal distribution’s mean value, its standard deviation value, and the estimated prevalence bias, three additional normal distributions were generated and studied. Their mean values were chosen to explore the signed pattern of biases around the critical value of -2.0 and include [-3.0,-2.0,-1.0]. Representing an ideal scenario of having no non-directional measurement errors, each distribution was generated to have an initial standard deviation of 1.0. Simulated non-directional error was then included by adding to each z-score a number drawn from a separate normal distribution with mean value 0.0 and standard deviation 1.0. Note that this non-directional error represents the total from all sources, including transcription, age, height, and/or weight measurement errors. By keeping track of which observations crossed the identification threshold of -2.0 as a result of this simulated non-directional measurement error, it is possible to study the relative rates of false positives and false negatives and ultimately the overall sign and magnitude of the bias in the final prevalence calculation relative to the initial ideal scenario.

The primary prevalence bias relationship result of this work assumes a z-score distribution free of non-directional error will have a standard deviation value of 1.0. But, genuine heterogeneity in childhood nutrition status across contexts will lead to a range of authentically diverse standard deviation values. Specifically, z-score distributions with standard deviation values in {0.8,1.2} are of plausibly high quality. So, similar to the exercise discussed previously the authors selected a few illustrative and relevant mean values and explored the relationship between their standard deviation and estimated prevalence bias with this more realistic scale in mind. For each mean value, the realistic range of biases is calculated by the difference in prevalence estimates between a given value of standard deviation and the prevalence values found at 0.8 and 1.2. In this way, instead of simple point estimates, these results provide a range of likely biases relevant to realistic scenarios.

To provide a use-case example of this prevalence bias relationship tool, the data from 21 socioeconomic surveys collected in the context of USAID-sponsored development activities were analyzed. The inclusion criteria was simply all datasets collected in the support of activity evaluation for an office in USAID available at the time of analysis. The datasets to be included in this analysis were determined prior to any examination of the included variables. Each survey used a multi-stage population-based sampling design and individual z-scores were weighted using values provided by the research teams. The prevalence bias relationship produced here was used to estimate the biases present in the height-for-age, weight-for-height, and weight-for-age z-score distributions for each survey included.

To investigate possible explanations for the observed data quality in these surveys, simple digit preference tests in the input distributions of age, height and weight were performed. Noting a likely signal present in the age distributions, the amount of age preference present in each survey was quantified by forming the ratio of observations found within the ages in the set 24 ± 1, 36 ± 1, and 48 ± 1 months divided by the total number of children included in the survey (hereafter referred to as the “age heaping ratio”). Ages near 12 months were excluded from this metric due to activity-specific eligibility requirements that treated children aged 0–18 months differently from those aged 18–60 months. Recognizing that this heaping near integer year ages is likely due to age estimation and therefore indicative of non-directional measurement error, a Pearson’s correlation value was calculated to test whether the strength of this variable is meaningfully connected to the inflated standard deviation values observed in the height-for-age distributions.

## Results

**Fig 1:**
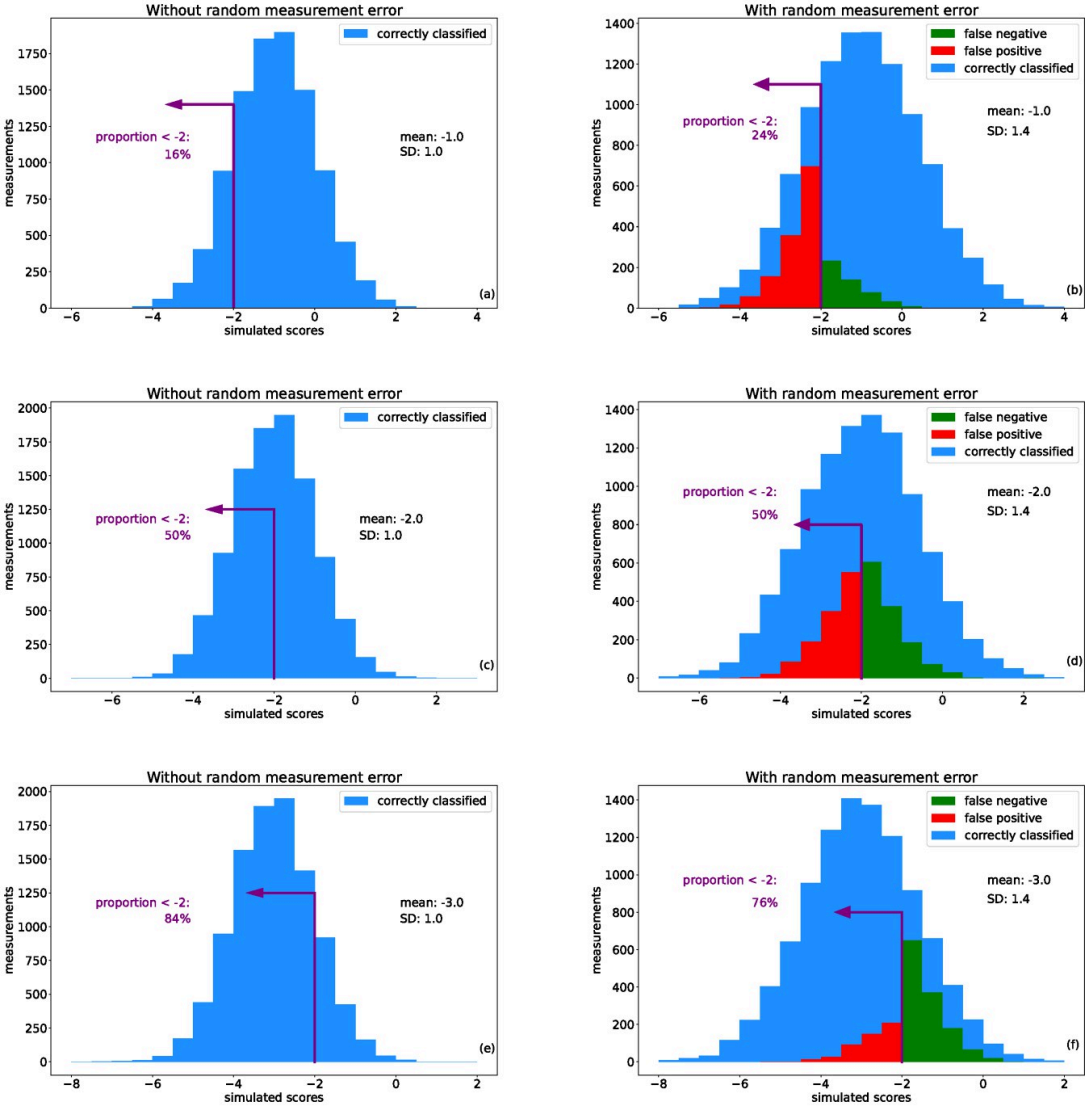
Simulated results for adding non-directional measurement error to three exemplary distributions. As labeled, panels (a), (c) and (e) are normal distributions generated with standard deviation 1.0 and mean values -1.0, -2.0, and -3.0, respectively. Panels (b), (d), and (f) show the result of adding non-directional measurement error. This demonstrates that for distributions with a mean value greater than -2.0, the false positive observations out-number the false negatives, leading to an overall positive bias in the measured prevalence rate (and vice-versa). In the special case of a distribution with mean value of -2.0, the two kinds of errors are balanced and no net bias is present.

The results of the case study with three normal distributions with mean values [-3.0,-2.0,-1.0] before and after the addition of simulated non-directional error are presented in Fig 1 Panels (a), (c), and (e) represent the ideal case of anthropometric z-score distributions for stunting, wasting or underweight having negligible non-directional measurement error and have a standard deviation of 1.0. Also printed on the figures is the quantity of interest in this work: the proportion of observations falling below -2.0. In these distributions with no simulated measurement error present, all observations are correctly identified which includes both true positive and true negative classifications.

The panels (b), (d), and (f) show the result of adding non-directional measurement error. Since the average value added to these distributions is zero, their mean values did not change but the standard deviation of each has increased from 1.0 to 1.4. Also overlaid in insets (b), (d) and (f) are the classification categories of false positive (that is, the observation’s value moved from above -2 to below -2 as a consequence of the simulated measurement error) and false negative determinations. The relative amounts of these two categories determines the overall bias in the prevalence calculation. That is, if the false positives occur at the same rate as false negatives, there will be no net shift in the calculated prevalence. However, for the distribution in panel (b), for example, it is clear that the false positives outnumber the false negatives. For this distribution the prevalence calculation has spuriously increased from the ‘true’ rate of 16% (shown in panel (a)) to 24% as a result of the added non-directional measurement error. Meanwhile, panel (f) shows an example of a distribution where the false negatives outnumber the false positives, leading to an underestimate of the prevalence (in this case, 76% compared to the true rate of 84%). This demonstrates that an overestimate or an underestimate is determined by the mean value of the original distribution relative to the critical value of -2.0. Inset (d) shows a distribution with the special case of having a mean value exactly at the critical value of -2.0. Here, the false positives are precisely balanced with the false negatives regardless of standard deviation, and the same prevalence rate independent of the added non-directional measurement error was found.

**Fig 2:**
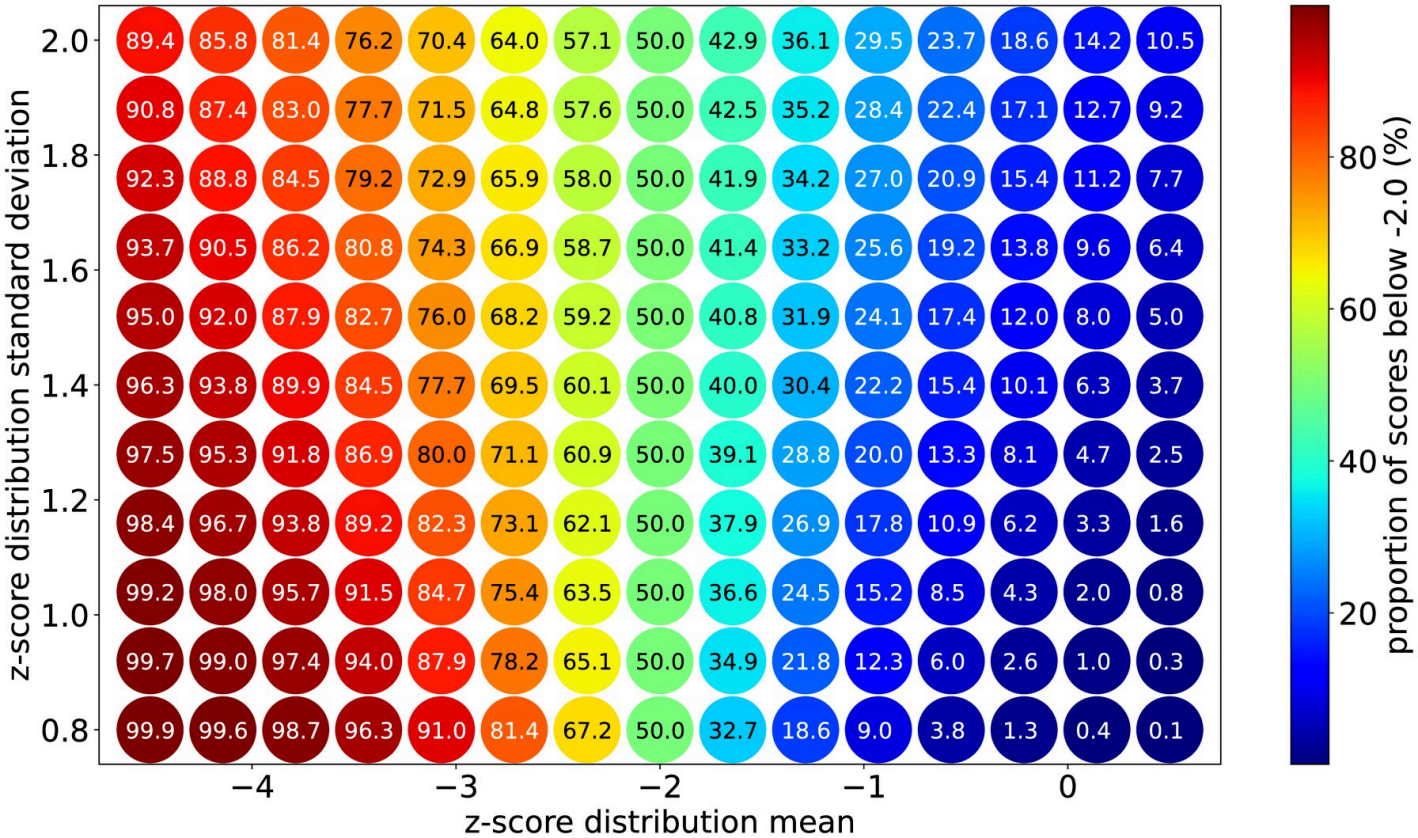
Relationship between a normal distribution’s mean, standard deviation, and prevalence of scores below -2.0.

The results of the full simulation campaign to describe the relationship between a normal distribution’s mean, standard deviation, and prevalence of scores below -2.0 for parameter values relevant to LMIC contexts is presented in Fig 2.

**Fig 3:**
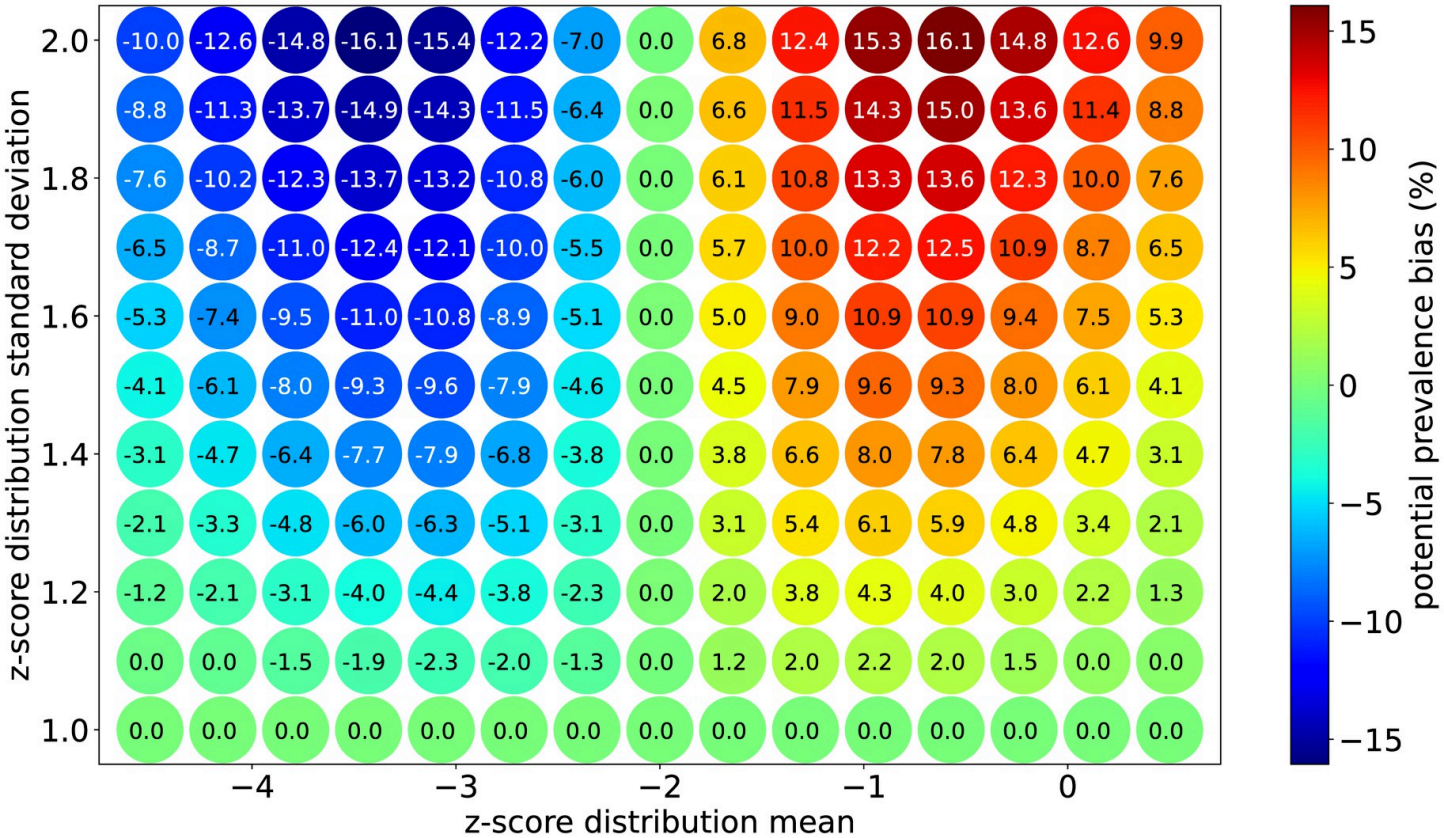
Estimated prevalence bias map.

Similar to the exercise summarized in Fig 1, under the assumption that the standard deviation should be close to 1.0 in the absence of non-directional error, the relationship shown in Fig 2 can be extended to estimate the bias in calculated prevalence rate for childhood nutrition indicators. Fig 3 presents the shift in prevalence for each set of distribution parameters compared to the ideal case of having a standard deviation of 1.0.

It is clear from the pattern shown in Fig 3 that the prevalence bias sensitivity to non-directional measurement error, via inflation of the standard deviation, depends non-linearly on the mean of the distribution. As shown earlier, prevalence rates for distributions with mean value -2.0 are insensitive to non-directional measurement error, while the sensitivity grows steeply for mean values on either side of -2.0. This sensitivity hits a maximum when the mean value is ±1.25 units away from −2.0 (i.e., −0.75 and −3.25) and moves towards zero farther from -2.0 in either direction. The prevalence bias is at a maximum overestimate when the mean value is ∼ −0.75 and at a maximum underestimate when the mean value is ∼ −3.25.

**Fig 4:**
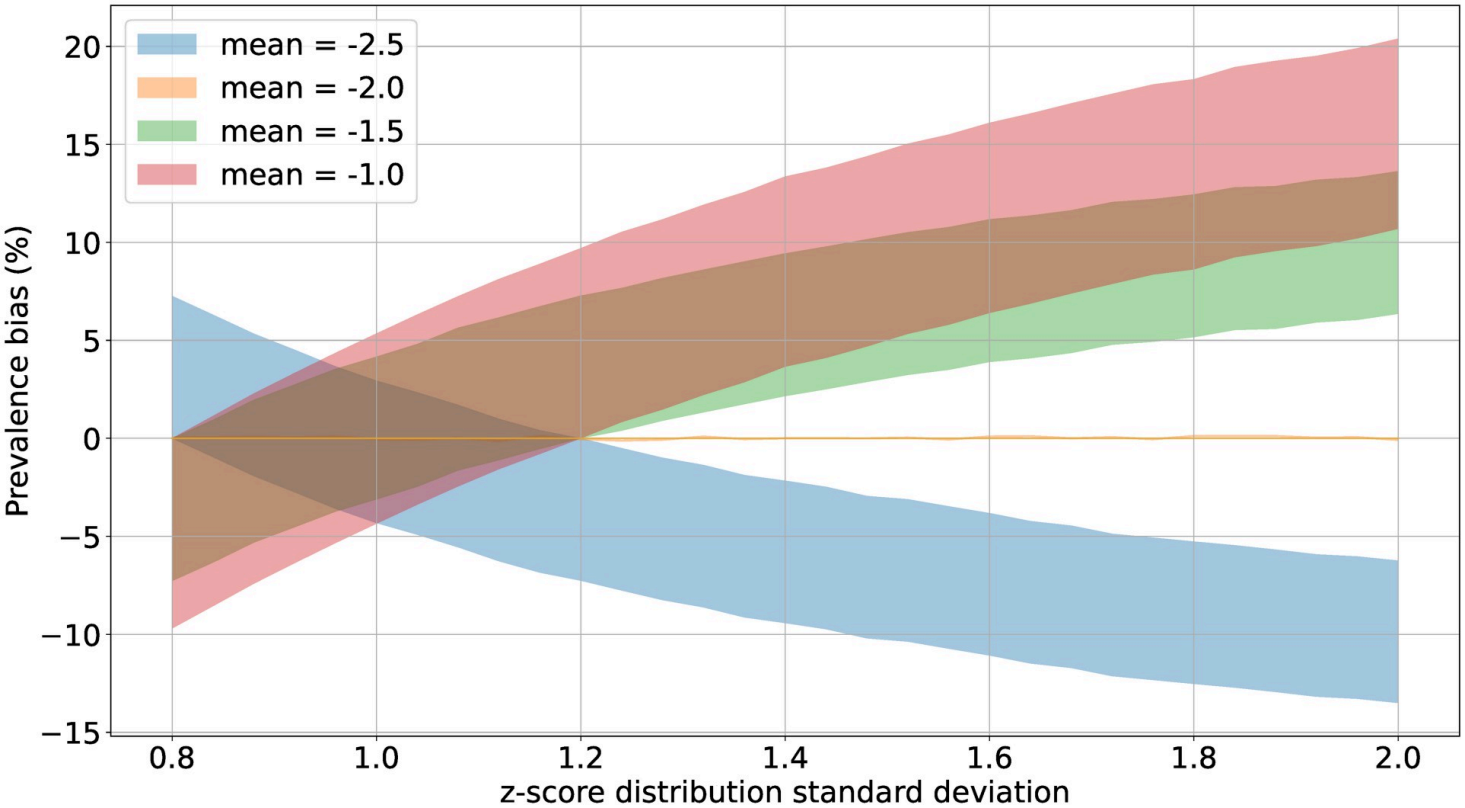
Scale of likely biases due to non-directional measurement error versus standard deviation value. A few exemplary mean values relevant to LMIC contexts are shown.

In order to use a more realistic scale of values to represent plausible nutritionally-heterogeneous populations, Fig 4 shows how the range of likely biases for some selected mean values depends on the distribution’s standard deviation value. As discussed in the Methods section, in this figure the contours are defined by the high-quality anthropometric data standard deviation reference range of 0.8—1.2. This complements the results in Fig 3 by showing these calculated biases may reasonably vary by 5% or more depending on the mean value and the genuine heterogeneity present in the community studied.

**Fig 5:**
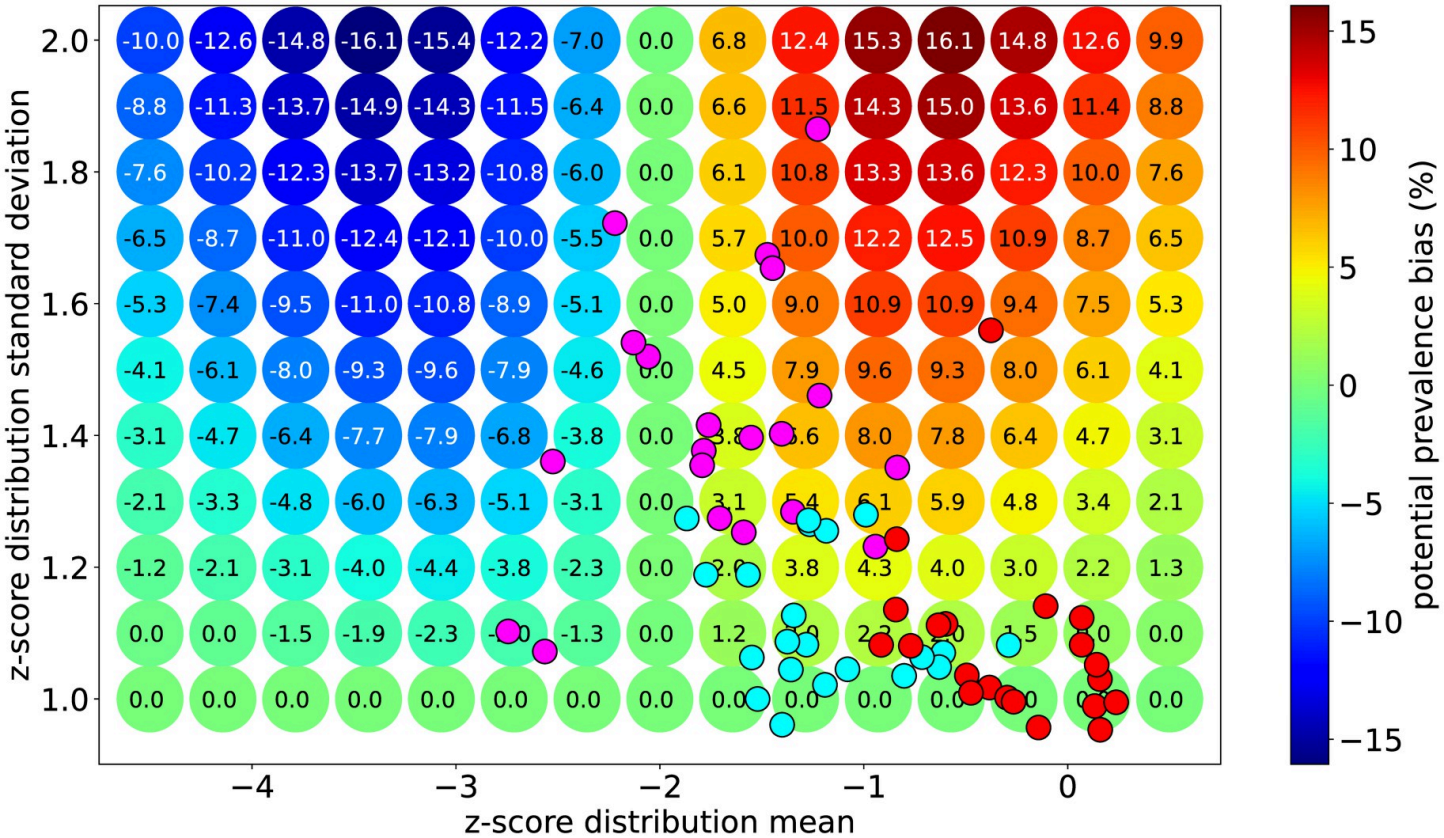
Scatter plot of mean and standard deviations overlaid on the estimated bias map. Included are the parameters for height-for-age (magenta), weight-for-age (light blue) and weight-for-height (red) distributions.

To demonstrate the efficacy of this tool, its results were applied to 21 child anthropometry survey datasets collected in LMIC contexts. Fig 5 overlays the weighted mean and standard deviation results of the three anthropometric z-score distributions for these 21 surveys on the bias scale results previously produced on Fig 3. This is particularly true for the height-for-age results, where only a minority of the estimated biases fall within typical confidence intervals of ∼ 2%. This dataset happens to be consistent with the trend observed in a systematic review of Demographic and Health Survey (DHS) data where the height-for-age standard deviations are generally higher than the values found in the weight-for-height or weight-for-age distributions [35] and are therefore more susceptible to biases in its calculated prevalence indicator of stunting. As a case-study on the details of this challenge, the rest of this section explores these data and also identifies the likely source of non-directional measurement error for this particular dataset. These data are available in S1 Dataset for replication and other analyses.

The clear majority of height-for-age distributions overlaid in Fig 5 have standard deviations that exceed 1.2, indicating likely significant presence of non-directional measurement error. An analysis of the input height and age distributions for these surveys was carried out with the intention to both potentially identify the source of the non-directional measurement error and also further build evidence supporting the hypothesis that the large observed standard deviations are in fact due to measurement error and not due to genuine heterogeneity within the surveyed populations.

**Fig 6:**
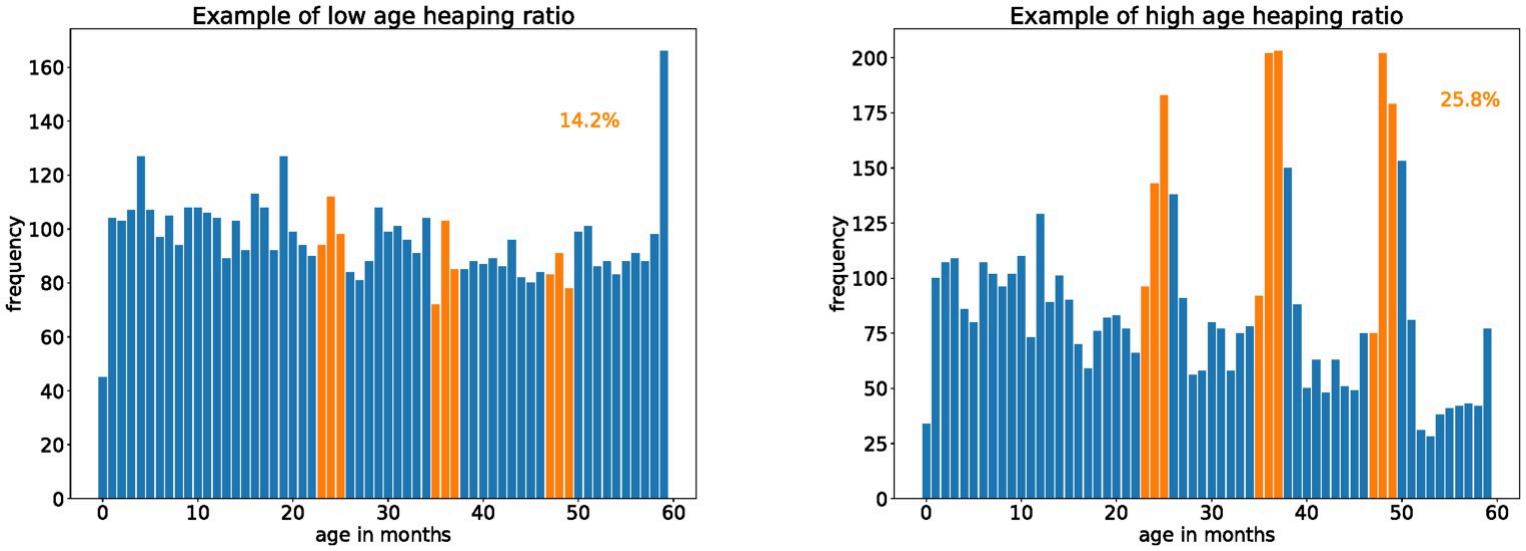
Examples of low (left panel) and high (right panel) age heaping ratios. This ratio is the fraction of observations at 24 ± 1, 36 ± 1, and 48 ± 1 (marked in orange) compared to the total.

No significant digit preference in the height distributions was observed. However, strong preferences were noted in the age distributions near the 24, 36, and 48 month positions. The age heaping ratio of observations within [23–25, 35–37, 47–49] months divided by the total quantifies the amount of children reportedly aged very near to 2, 3 and 4 years of age. Note that, if the children’s ages were uniformly distributed, this quantity would be 9/60 = 15%. Fig 6 shows examples of age distributions with relatively low and high age heaping ratios.

**Fig 7:**
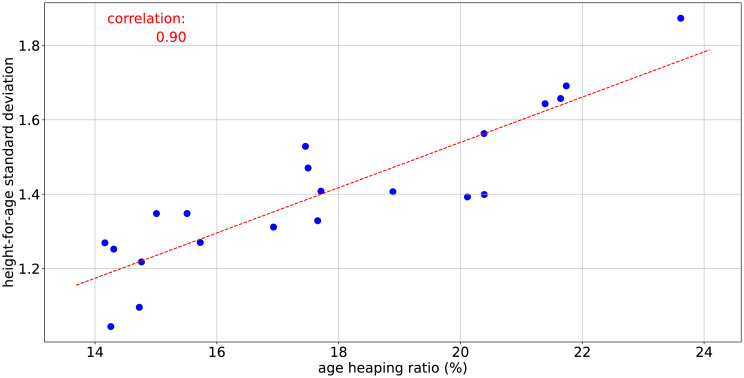
Scatter plot of height-for-age standard deviation versus age heaping ratio.

Following the Pearson’s correlation test, a surprisingly strong relationship (*ρ* = 0.90) was found between the age heaping ratio and the standard deviation of the height-for-age distributions. Fig 7 shows the scatter plot for these data.

This finding demonstrates that the large standard deviation values amongst these surveys are very likely due to age measurement error and strongly disfavors the hypothesis that they are due to genuine height-for-age heterogeneity within the community. This is consistent with the expectation that anthropometric distributions with low non-directional measurement error have standard deviation values dominantly between 0.8 and 1.2. It also indicates that, while height measurement challenges are not excluded from contributing, the age-related data quality is the clear driving factor in the accuracy of the stunting indicator for these datasets. Combined with the earlier demonstrations that non-directional measurement error can lead to erroneous and significant prevalence indicator shifts, this provides strong evidence that the stunting prevalence rates produced from the included surveys may be significantly biased and the tool created here should be used to estimate its severity.

## Discussion

This work simulates the effect of non-directional measurement error for threshold-based prevalence indicators for normal distributions. The three-way relationship between a normal distribution’s mean value, its standard deviation, and prevalence below -2.0 was explored and presented. Together with knowledge that a childhood nutrition z-score distribution’s standard deviation value should be close to 1.0 in the absence of non-directional measurement error, a prevalence bias map (Fig 3) was produced. In general, the higher the standard deviation, the more severe the prevalence bias will be. However this dependence is also highly sensitive to and interacts with the z-score distribution’s mean value. For a given standard deviation greater than 1.0, the prevalence will be maximally *overestimated* when the mean is ∼ −0.75 and maximally *underestimated* when the mean is ∼ −3.25. Between these values, the bias is zero at the special value of -2.0 regardless of standard deviation. The greatest vulnerabilities to prevalence bias occur when the z-score distribution has a large standard deviation *and* whose mean value is near −3.25 or −0.75. So, if a given z-score distribution’s mean and standard deviation value are accessible, these findings can be used to understand the degree to which any contemporary or historical survey’s anthropometric prevalence results are vulnerable to biases due to non-directional measurement error. It should be noted that these results are entirely independent of the ultimate source of the non-directional measurement error. That is, the calculation made no assumptions on whether the errors came from issues in transcription, height, age, or weight measurements and therefore the findings are valid across broad contexts.

Unfortunately, it is common to find only the childhood anthropometry prevalence results reported in the survey dissemination efforts and no additional information about the underlying z-score distributions. As part of regular quality control and transparency practices, at a minimum the z-score mean and standard deviation values should be reported along with the primary indicator results.

As a use-case example, the prevalence biases of a dataset of childhood anthropometry collected in LMIC contexts were estimated and their data quality was examined. In this case, of the three indicators studied, the stunting indicator happened to suggest the most severe biases with some exceeding 10%. As the confidence intervals associated with these surveys generally do not exceed 2%, the majority of the estimated biases would significantly change the interpretation of these surveys. A simple data quality check revealed no systematic issues in either the weight or height input distributions, but a surprisingly strong relationship was found between age heaping and the height-for-age distribution’s standard deviation. This suggests age data quality is plausibly the main driver for the overall quality of the stunting indicators calculated in this dataset, and that the elevated standard deviations observed are indeed due to measurement error and not a genuinely heterogeneous survey population. For the kind of community development programs implemented in the context of these surveys, a typical target for stunting reduction is one percentage point per year. Over a typical programmatic length of five years, it is clear from the steep sensitivity shown in Fig 3 that differential biases between baseline and endline surveys may partially or even completely obscure the ability to detect change consistent with this target reduction. Note that changes in mean value are *expected* when development programming aims to affect childhood nutrition at a community level. Therefore even if the data quality and community heterogeneity were static between baseline and endline surveys, real changes in the distribution’s *mean* are likely to result in differential indicator biases that threaten the validity of the findings.

In parallel to efforts to understand the effects of measurement error on childhood nutrition indicators such as the work presented here, it is important to also pursue methods of improving the quality of data directly. From the authors’ perspective, the following practices are among the most likely to contribute to good and consistent data quality in the context of LMIC child anthropometry surveys:

- Include a standardization exercise as part of survey training and allow a minimum of one full day of intensive anthropometrist training.
- When supporting multiple rounds of surveys aimed at detecting change, maintain the same field survey organizations using the same high standards of best practices across surveys.
- When possible, confirm age using health cards to avoid age estimation and therefore reduce age heaping.
- When health cards are not available, use local calendars and/or notable events such as religious holidays or remarkable weather to reduce age estimation.
- Always confirm the biological sex of child with a caregiver before measuring.
- Always take height and weight measurements twice, and keep measuring if the difference in the recorded values differs more than a pre-determined error threshold until consistency is reached.
- Perform both systematic and random quality assurance checks of enumerators including unplanned supervision during data collection and calculating determine inter- and intra-enumerator differences in measurement values.
- Perform frequent refresher anthropometry training events on anthropometry across sex and age groups.
- Always calibrate anthropometry equipment with items of a known weight or length before and after use and replace faulty equipment.
- Implement a checklist for measuring children that captures potential confounding factors and differs for infants (≤ 24 months) and older children (24–60 months). The infant checklist may include:
  - Is the infant undressed? If infant is wearing a diaper during assessments, check to be sure it is clean and tare the scale using a diaper of similar weight before weighing the infant.
  - Does the infant have a hairstyle that is significantly contributing to height?
  - Is the infant wearing clothes that are significantly contributing to weight?
- The checklist for older children could contain:
  - Has the individual removed their shoes? Jewelry? Hair accessories? Outer layers?
  - Does the individual have a hairstyle that is significantly contributing to height?
  - Is the individual wearing clothes that are significantly contributing to weight?
  - Is the individual standing with their heels against the wall?
  - Has the individual removed clothing covering their arm for MUAC assessment?

It is important to note again that this work does not address the potential presence of *directional* measurement errors in children’s anthropometric data. As the technique discussed in this work relies on the accuracy of the distribution’s mean value, the authors strongly recommend against using the bias estimate as a *correction* to the survey’s primary findings. Instead, the authors encourage researchers to use this estimate as an additional tool to evaluate their data quality by finding and reporting the rough sensitivity of their childhood anthropometric findings to non-directional measurement error. This would represent an improvement to survey rigor, promote data quality transparency and allow diverse stakeholders including policy decision makers to better understand the state of and trends in global childhood nutrition.

## Supporting information

S1 Dataset Anthropometry data for the 21 surveys used in the data quality assessment. Included are the child’s age in months (variable name ‘age’), z-score values for height-for-age (‘haz’), weight-for-height (‘whz’), weight-for-age (‘waz’) and survey weights (‘wt’).
(ZIP)
